# Coworking Spaces: A Source of Social Support for Independent Professionals

**DOI:** 10.3389/fpsyg.2016.00581

**Published:** 2016-04-25

**Authors:** Cornelia Gerdenitsch, Tabea E. Scheel, Julia Andorfer, Christian Korunka

**Affiliations:** ^1^Department of Applied Psychology: Work, Education, Economy, Faculty of Psychology, University of ViennaVienna, Austria; ^2^Social and Organizational Psychology, Department of Psychology, Faculty of Life Science, Humboldt University of BerlinBerlin, Germany

**Keywords:** coworking space, new ways of working, social support, entrepreneurship, ressources, social interaction

## Abstract

Coworking spaces are shared office environments for independent professionals. Such spaces have been increasing rapidly throughout the world, and provide, in addition to basic business infrastructure, the opportunity for social interaction. This article explores social interaction in coworking spaces and reports the results of two studies. Study 1 (*N* = 69 coworkers) finds that social interaction in coworking spaces can take the form of social support. Study 2 further investigates social support among coworkers (*N* = 154 coworkers) and contrasts these results with those of social support among colleagues in traditional work organizations (*N* = 609). A moderated mediation model using time pressure and self-efficacy, based on the conservation of resources theory, is tested. Social support from both sources was positively related to performance satisfaction. Self-efficacy mediated this relationship in the employee sample, while in the coworking sample, self-efficacy only mediated the relationship between social support and performance satisfaction if time pressure was high. Thus, a mobilization of social support seems necessary in coworking spaces. We conclude that coworking spaces, as modern social work environments, should align flexible work infrastructure with well-constructed opportunities for social support.

## Introduction

Coworking spaces are shared office environments for independent professionals (Pohler, 2012; Spinuzzi, 2012) and have been increasing rapidly. In 2015, 7,800 spaces existed worldwide with a growth rate of 83% from 2012 to 2013 (Foertsch, 2013) and of 36% from 2014 to 2015 (Foertsch, 2015). Various professionals, predominantly independent ones such as freelancers or remote workers, use these spaces as their places of business (Pohler, 2012). Most of these independent professionals worked from home prior to renting a place of work in a coworking space, where they may have suffered from feeling of isolation, among other problems (Spinuzzi, 2012). Thus, coworking spaces represent one possible buffer against isolation by providing, in addition to business infrastructure, the opportunity for social interaction.

This paper reports the results of two studies. Within the first, we explore social interactions in a coworking space (Study 1) and question whether they can take the form of social support. Social support describes an exchange of resources between at least two persons with the intention to help (House et al., 1988). Aspects of social support are direct support, affective support, or confirmation (Kahn and Antonucci, 1980; House et al., 1988). Besides examining whether social support is a reality in coworking spaces, we investigate the effect of social support in a second study (Study 2). We focus on work-related social support (Frese, 1989), which has been widely studied in the context of traditional workplace settings. Within the second study, we particularly contrast the effect of social support from coworkers in these new spaces with that from colleagues in traditional workplace settings. We assume that coworkers in a coworking space may represent a new source of social support for independent professionals, operating along different lines than colleagues and supervisors in a traditional setting. We propose a moderated mediation model of social support, which we expect to be valid for both the coworking and employee samples. In particular, we expect a positive relationship of social support to satisfaction with performance, mediated by self-efficacy (see also Rhoades and Eisenberger, 2002; Osca et al., 2005; Bakker and Demerouti, 2007). Further, we assume that this mediation is moderated by time pressure. We expect that support will be activated when there is high time pressure, which strengthens mediation in both the traditional and coworking settings.

Although there has been an increase in the number of coworking spaces, scientific research has still not paid adequate attention to this emerging office environment. Qualitative research articles (Pohler, 2012; Spinuzzi, 2012; Garrett et al., 2014), case studies (Fuzi, 2015; Orel and Rus, 2015), unpublished work (Foertsch, 2013), and blogs have described the characteristics of coworking spaces, the people who work there, reasons for working there, and how and when a sense of a community emerges in these spaces. However, to the best of our knowledge, there is no published empirical quantitative research article describing coworking spaces and no study that explores social interactions and the effects of being supported by other coworkers in coworking spaces. This article contributes to research in this area in two ways.

First, within Study 1, we explore social interaction in a coworking space, considering the question of whether social interaction takes the form of social support. We thus extend existing research on physical office environments (Elsbach and Pratt, 2007; Ashkanasy et al., 2014) by investigating coworking spaces as emerging new office environments. Second, existing research on social support in the work context has mainly focused on social support from colleagues and supervisors (Baruch-Feldman et al., 2002). We now argue that for this specific sample of independent professionals, alternative sources of social support may become more relevant and may be more appropriate given that colleagues and supervisors are either not consistently available or non-existent. Thus, our study extends the research on social support by investigating its presence in a new population: coworkers in a coworking space.

## Study 1: Social Interactions in Coworking Spaces

Professionals in coworking spaces have flexibility regarding their work location and are thus described as the prototypes of the “boundaryless workforce” (Pohler, 2012). Such location flexibility comes with some obstacles. One is professional isolation (Vega and Brennan, 2000; Bailey and Kurland, 2002), which “occurs when the desires for support, understanding, and other social and emotional aspects of interaction are not met” (Taha and Caldwell, 1993, p.277). In line with a recent article (Spinuzzi, 2012), we argue that coworking spaces provide a potential solution to professional isolation, since they aim to establish a social atmosphere in which social interaction and collaboration are possible (Pohler, 2012; Moriset, 2014).

Social interactions in coworking spaces may come in various forms. On the one hand, people may simply work alongside each other or engage in rather casual conversation. On the other hand, coworkers may engage in networking, seek and obtain feedback, share ideas, or collaborate (Spinuzzi, 2012). However, very little is known about what social interaction in coworking spaces looks like. Rather, it is still unclear whether social interaction in a coworking space takes the form of social support, as it often does between colleagues in traditional work settings.

Basically, social interaction is a process by which people act and react to those around them (Giddens, 2009). Social interactions take various forms that can be positive and/or negative. The simple presence of others or the fact of working with others rather than alone can for instance be positive, although it does not yet represent socially supportive interactions. Social interactions that are beneficial to one or both parties qualify as social support (Shinn et al., 1984). In particular, social support describes an exchange of resources between at least two persons, whereas the sender who provides support aims to help the person who receives the support (House et al., 1988). Three aspects of social support are identified (Kahn and Antonucci, 1980; House et al., 1988): direct support (instrumental support, exchange of information), affective support (admiration, liking), and confirmation about actions and statements.

Coworkers are independent workers and are thus not as closely aligned as regular colleagues because they are usually not working toward the same goal. There is also no task interdependence between them. They may even be in direct open competition with one another if they work in a space that serves people with a specific specialization or field of work. However, there are theoretical arguments that make us assume that social support is a significant reality in coworking spaces. In reference to the social identity approach, individuals classify themselves as members of social categories to locate themselves within a given environment (Tajfel and Turner, 1986; Asforth and Mael, 1989). Coworkers may define themselves as part of the global coworking community group and/or as part of their specific workspace group. Members of a group typically act in a way that supports the other members of this group (Asforth and Mael, 1989).

There are some indications that coworkers indeed identify at least to some extent with the global coworking community and/or the specific coworking space where they are working. People working in coworking spaces are part of the global coworking community, which is interconnected via diverse media. For instance, a Coworking Wiki lists coworking spaces worldwide and a definition about coworking spaces. This definition highlights that coworking spaces are more than mere shared offices; coworkers share the same core values: collaboration, openness, community, accessibility, and sustainability^1^. Furthermore, conferences (global coworking unconference conference; coworking Europe conference) bring together people of the community. On the level of the coworking space, “hosts” or “community managers” organize events to strengthen exchange and community thinking. Garrett et al. (2014) also describe how a sense of community emerges in coworking spaces.

To sum up, the coworking movement includes some sort of group thinking. Being part of the same social group promotes supportive behavior and makes it easier to ask coworkers to listen to job-related as well as personal problems. Which may foster supportive behavior. The aim of Study 1 is to explore the nature of social interactions that take place in coworking spaces. We expect that such social interactions can take the form of social support.

### Materials and Methods

#### Sample and Procedure

The sample consisted of 69 coworkers (average age = 32.02, *SD* = 5.99) working in eight different coworking spaces in Austria (45 male). Most (80%) held a university degree. The sample was international, with most participants being Austrian (51%), followed by Germans (16%), and 18 other nationalities (30%; 3% missing). We recruited the sample by personally promoting our study in coworking spaces in Austria and through social media. The survey was available both online and in paper–pencil format.

#### Measures

The survey consisted of sociodemographic questions (gender, age, tenure, nationality, education, and employment status) and questions pertaining to social interaction with other coworkers. Specifically, we asked participants to describe situations in which they interacted with other coworkers. This approach of a retrospective self-report about specific situations is similar to the critical incidence technique (Flanagan, 1954; Butterfield et al., 2005) and enables us to gather rich information about social interaction from the participants’ perspectives. To ensure responses that describe a widespread variety of situations, we instructed participants to think of three situations, a casual/short interaction, medium-length interaction, and a longer interaction. The question was worded as follows: “*Please think of 3 situations in your coworking space when you interacted with coworkers, one situation with a short/casual social interaction, one with a medium length, and one situation with a long/intensive social interaction. Please briefly describe the situations in the following paragraphs (*∼*5 sentences each).”*

#### Analyses

We applied a summarizing qualitative content analysis (Mayring, 2008) to cluster the situational descriptions. The first step entailed reformulating original statements into a content-related linguistic form (paraphrase). Three raters discussed the original statements and came up with a set of categories that describe the essence of the original statements. Subsequently, another independent rater was asked to categorize the statements deductively.

### Results

We collected 178 descriptions of social interactions in coworking spaces (65 short, 58 medium, 55 long). Content analysis resulted in four categories representing the descriptions. Two represent the two aspects of direct social support that are instrumental, support and exchange of information. The others describe informal social interaction and collaboration within projects. Cohen’s κ was run to determine agreement between the raters. There was a good degree of agreement (κ = 0.744, *p* < 0.0005) according to the threshold of 0.7 suggested by Landis and Koch (1977). More precisely, raters agreed with regard to the category “informal social interactions” in 74 (out of 85), “exchange of information” in 33 (out of 41), “instrumental support” in 24 (out of 30), and “collaboration” in 16 (out of 22) situations. The categories are described below.

*Informal social interactions* (85 statements). On a basic level, coworkers reported encounters when they greet other coworkers and have short conversations over coffee or cigarettes and over lunch. Two coworkers described the following situations. “After coming to the office in the morning, I usually get some coffee. Other people are frequently passing by on their way in, so we typically have a short chit-chat” (#11, 31 years, male); “Was grabbing a coffee, met a coworker. Just briefly introduced ourselves. Waited for his coffee to finish (coffee machine - > cup) as well. Said goodbye” (#53, 22 years, male). These situations can also get deeper when coworkers have personal conversations about life while for instance going for drinks after work or doing sports together. One participant described the following situation. “We were working late, and I didn’t care to go home. I asked a colleague to go for a beer. We ended up talking for several hours and covered personal, work, and emotional topics” (#20, 25 years, male).

*Exchange of information* (41 statements). In contrast to informal social interactions, this category includes social interactions that are explicitly work-related. Coworkers describe work-related conversations with other coworkers, but also their engagement in official networking activities in their coworking space. During lunchtime and coffee breaks, coworkers get to know the projects other coworkers are working on. “When people have lunch, we often sit and eat together while talking about projects, tech-related things (with other engineers) or whatever comes to mind. Most people know each other well enough that having lunch together is not awkward” (#11, 31 years, male). Besides updating each other on current projects and networking, coworkers also reported discussing potential collaborations or planning common activities (workshops). They also reported attending events or workshops organized in their coworking space: “I went to the AngularJS meetup. Listened to a couple of interesting lectures, met some interesting people. It was a good opportunity to meet people with similar interests and learn a few things” (#7, 30 years, male).

*Instrumental support* (30 statements). Coworkers reported asking for or providing help in terms of feedback, brainstorming, and coaching. In contrast to information exchange, these statements are about situations in which workers report helping each other in a concrete task. With regard to feedback, coworkers reported asking for feedback or providing support for problems. Examples of such feedback situations are the following: “A coworker asked me for my opinion on some websites he was designing” (#36, 35 years, male); “I asked a coworker about a technical problem. He/she took 10 min of time to listen and propose a solution” (#49, 53 years, male); “I was having a lunch session with a coworker where I gave feedback on project idea” (#17, 30 years, male). Furthermore, coworkers asked others to engage in short brainstorming sessions, which can also take the form of coaching. Three coworkers described the following situations: “I had a 1-h chat over coffee with a member of the community about his next steps, as he’s at the moment standing on the crossroad of opportunities (changing career, discovering where he wants to go); I also shared an idea with him about starting” (#62, 25 years, male); “Business modeling support for a coworker’s start-up company, strategy to apply for public grant, strategy for talking to external investors/business angels, contact with and meetings with business angels” (#25, 43 years, male); “Meeting with a coworker every 14 days for mutual coaching and exchange, for the past 2 years” (#51, 45 years, female).

*Collaboration* (22 statements). Besides providing feedback, brainstorming, and coaching, coworkers also engage in collaborations with one another, both paid and unpaid. They reported working together on an idea or ask others to take over some tasks. One described recruiting someone in the space to do some paid work: “A professional article was needed for a huge online magazine and a PR/marketing expert coworker helped us to write it” (#63, 33 years, female).

### Brief Discussion of Study 1

Study 1 improves the understanding about social interactions that occur in a coworking space. Coworkers describe most of the situations as informal social interactions. We suggest that those social interactions enable coworkers to create a social network. Interestingly, we also found that collaboration between coworkers is possible. In these situations, people working in coworking spaces managed to create synergies or common benefits between their own businesses in contrast to competing with each other. Finally, our findings showed that situations of supportive behavior indeed take place in coworking spaces. The categories “instrumental support” and “exchange of information” represent aspects of direct social support (Kahn and Antonucci, 1980; House et al., 1988). Within Study 2, we aim to shed light on this form of social interactions. Work-related social support has been researched as one relevant job resource in traditional workplace settings. The question remains if effects of social support from coworkers are similar to effects from colleagues in a traditional office setting.

Limitations to the study involve possible self-selection of participants due to the research approach. Coworkers who are more integrated in their coworking space may be more willing to participate in such a study and more likely provide social support. However, we do not conclude that every coworker engages in social interaction, but demonstrate that social support does indeed occur in coworking spaces. Thus, a coworking space represents a social environment that provides social support, representing one important job resource.

## Study 2: Social Support in Coworking Spaces

In the first study, we described that social interactions in coworking spaces take the form of direct social support, but the effects of this social support are still unclear. While several studies have described beneficial effects of support from colleagues and supervisors on work stress (mitigated stressors, reduced strain, buffer between stressor and strain; meta-analysis by Viswesvaran et al., 1999) and job satisfaction (supervisor support leads to various beneficial outcomes mediated by perceived organizational support; meta-analysis by Rhoades and Eisenberger, 2002) in traditional office settings, no study has investigated the effects of social support from coworkers in a coworking space.

**FIGURE 1:**
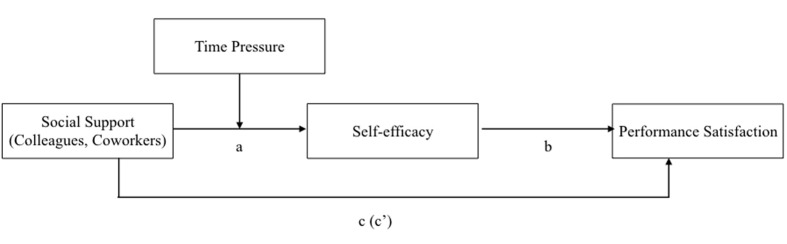
**Proposed research model**.

The aim of Study 2 was to contrast the effects of social support in a traditional office and a coworking space setting. We expect to see similar beneficial effects of social support from coworkers as we do from colleagues in a traditional work setting. We collected two samples, one in traditional office settings (colleagues as source of social support) and the other in coworking spaces (coworkers as source of social support) to contrast the effects of social support from coworkers and from colleagues. Based on the conservation of resources theory (COR theory; Hobfoll, 1989, 2002), we derived a moderated mediation model that we tested for both sources of social support. The model is depicted in **Figure 1**. The specific hypotheses are described in the following sections.

A considerable amount of literature has been published on the main and moderating effects of social support in the working context (Cohen and Wills, 1985; Viswesvaran et al., 1999; Rhoades and Eisenberger, 2002). Several studies found positive effects of social support on performance (Searle et al., 2001; Baruch-Feldman et al., 2002; Osca et al., 2005) and goal achievement (Bakker and Demerouti, 2007). In particular, social support can help employees feel good about themselves, leading to a positive evaluation of their performance (Searle et al., 2001). In line with this research, we assume that experiencing social support from colleagues positively affects performance.

For people working in coworking spaces, we expect to find the same effect of social support. Spinuzzi (2012) argues that before visiting a coworking space, coworkers often suffer from professional isolation, which has a negative effect on performance that can be buffered by, for example, more face-to-face interaction (Golden et al., 2008). Therefore, we argue that coworkers will benefit from social support in terms of performance. Since the population of coworkers is diverse with respect to their businesses (Pohler, 2012), a comparison of objective indicators of performances is difficult, if not impossible. Therefore, we used self-rated performance satisfaction as a proxy measure for objective job performance.

*Hypothesis 1*: We expect that social support from (a) colleagues and (b) coworkers is positively related to self-rated performance satisfaction.

Resource models propose that resources are linked to each other, rather than existing in isolation (Hobfoll, 2002). In this vein, the beneficial effects of social support on work-related outcomes can be explained by a joint and reciprocal activating effect of resources. COR theory (Hobfoll, 1989, 2002) explains how resources relate to each other. This theory suggests an enrichment of resources, a so-called resource gain process (Hobfoll, 1989), whereby the existence of one resource facilitates the development of other resources (Hobfoll, 2002). As such, social support (one resource) can clear doubts about one’s competence and thus increase self-efficacy (another resource). Self-efficacy describes the belief in one’s ability to master challenges. It can be enhanced by social persuasion from significant others (Bandura, 1977). The role of self-efficacy has been confirmed in several studies as a mediator (Luthans et al., 2008; Mastenbroek et al., 2014) of the relationship between job resources and work-related outcomes.

We expect that for employees as well as for coworkers, social support activates self-efficacy in such a way that workers have more faith in their own abilities to successfully master work-related challenges (increased self-efficacy). As a consequence, both employees and coworkers should be more satisfied with their performance.

*Hypothesis 2*: We expect that self-efficacy mediates the relationship between social support from (a) colleagues and (b) coworkers and performance satisfaction.

Conservation of resources theory suggests that resource gain cycles are most likely to emerge during highly stressful circumstances (Hobfoll, 1989, 2002). The more stress, the more likely workers are to seek out or receive social support (support-mobilization hypothesis, Eckenrode, 1983; Stephens and Long, 2000). Such a mobilization or activation of social support can again facilitate the development of new resources. However, a meta-analysis of employee data found no evidence for the support-mobilization hypothesis in the context of work stress (Viswesvaran et al., 1999). In the present study, we test this hypothesis in the context of performance. Drawing on COR theory, we expect that social support will be activated when time pressure is high, which will further facilitate development of self-efficacy.

We argue that such an activation of social support is also relevant for professionals in coworking spaces. In contrast to employees with colleagues and supervisors in traditional office contexts, no predefined person is available for work-related questions in a coworking space. Coworkers have to actively create and build a social network, and stressful situations with high time pressure can potentially stimulate support seeking. We further expect that when social support is activated due to time pressure, the beneficial effects on satisfaction with performance via self-efficacy will also be strengthened (Bakker and Demerouti, 2007). Therefore, we formulated the following hypothesis.

*Hypothesis 3*: We expect that time pressure moderates the mediating effect of self-efficacy on the relationship between social support from (a) colleagues and (b) coworkers and performance satisfaction such that the mediating effect will be stronger when time pressure is high.

### Materials and Methods

#### Sample and Procedure: Colleagues

The employee sample consisted of 609 employees (330 female; average age = 29 years, *SD* = 3.90). In all, 35% obtained a university degree, and most of the coworkers worked full-time (90%). On average, they had worked 46.85 months (*SD* = 38.14) in their respective organizations. More than half of the sample consisted of Germans (53%), followed by Austrians (45%; 2% remaining). The sample of employees was gathered via an ISO-certified (ISO 26362) German online panel^2^, which ensures high quality data by minimizing participation frequency and conducting continuous controls.

#### Sample and Procedure: Coworkers

The coworking sample consisted of 154 coworkers across Europe (102 male and 52 female). Their average age was 39 years (*SD* = 8.45). Most participants (79%) had a university degree, and 12% had a high school degree. Gender, age, and the highest educational level of the sample were comparable to the non-scientific second annual coworking survey by Deskmag (*N* = 1532; Foertsch, 2012). In this study, the gender distribution was 66% male, 34% female, the average age was 34, and 75% of the coworkers held a university degree. Participating coworkers had worked in a coworking space for an average of 18.1 months (*SD* = 22.2), whereas they spent on average more than half of their working time (65%) in a coworking space. Altogether, the sample consisted of coworkers from 52 different coworking spaces from 17 countries, located in 37 cities. Participants included 24 nationalities, with the majority being Austrian, Portuguese, and German (see **Table 1**).

**Table 1:** Sample characteristics of the coworking sample.

Employment status^a^	*%*	Nationality	Frequencies
Self-employed	62	Austrian	50
Freelancer	35	Portuguese	20
Full-time employees	16	German	18
Part-time employees	9	Italian	10
Student	8	French	8
Other occupational contracts	7	Czech	5
		
Frequency of using a coworking space^a^	*%*	Polish	5
		
Full-time	74	American	4
A few hours a day	29	Slovenian	4
Sporadically	12	British	3
On weekends	11	Bulgarian	3
At night	10	Hungarian	3
		
Other time preferences	8	Spanish	3
		
Occupation	Frequencies	US	2
Software/web development, design	27	Dutch	2
Consultancy, management	16	Mexican	2
Writing, journalism, blogging, language services	10	Brazilian	2
Science, research/technology, education	7	Others	8
Online (social media) marketing/communication, PR	7	Missing	2
		
Working for a space	5	Reasons^a^	%
		
Design, creative projects	5	Social interaction	83
Arts, architecture	3	Productivity	68
Social entrepreneurship	4	Networking	67
Tourism, gastronomy	2	The provision of infrastructure	66
Others	9	Flexibility	63
		
Missing	4	Workplaces used^b^	*Mean (SD)*
		
		Coworking space	64.47 (29.05)
		Home office	25.27 (24.19)
		Other third places (café, on the move, etc.)	12.96 (13.19)
		One’s own office	8.87 (20.73)
		A friend’s office	2.49 (7.21)

*^a^Multiple options possible. ^b^Reported means and standard deviations are related to the percentage of the respective working time at the different places*.

Participants were recruited using three strategies. First, we contacted the managers of coworking spaces and asked them to distribute the online survey link to coworkers in their coworking space and to send a reminder. To give potential participants an understanding of the study and to motivate them to take part, we prepared a self-made recruiting video. Of a list of 208 coworking spaces in 27 European countries from the Coworking Wiki^3^, 50 spaces guaranteed their support. Second, we contacted journalists from relevant online magazines (Deskmag, Coworking News) and asked them to post the link to the survey across a diverse array of social media and to write blog entries about the study. Finally, the study was personally promoted in coworking spaces in Vienna. Due to the international sample, the online survey and all recruiting materials were in English.

#### Measures

We used the same measures for the sample of employees and coworkers. To verify the adequacy of the survey for the sample of coworkers, we conducted a group discussion with three members of a coworking space in Vienna (Austria) prior to the start of the study. The three members were a male (28 years) software developer, a female (30 years) owner of a social media marketing agency, and a male (25 years) mobile app developer. They filled out the first version of the survey, and we discussed the questionnaire’s items sequentially with regard to their comprehensibility and appropriateness for the target group of coworkers. The survey was modified to its final version taking their feedback into account. In particular, the two items for measuring performance satisfaction were created in consultation with these members. The following variables (except the questions about characteristics of the coworking sample) were used to describe the working conditions of both employees and coworkers.

##### Social support

Social support was measured with the Work-Related Social Support Scale (SzSU, (Frese, 1989). Answers to the five questions were rated from 1 (*not at all*) to 4 (*completely*). An example item from this scale is: “How willing are these persons to listen to your problems with your job?” In answering the questions, employees were instructed to refer to colleagues and coworkers to other coworkers.

##### Time pressure

Time pressure was assessed using a four-item subscale from the Instrument for Stress-Related Job Analysis (ISTA, Semmer et al., 1998). The items were scored on a scale ranging from 1 (*very rarely/never*) to 5 (*very often*). An example item is: “How often do you have to work faster than normal in order to complete your work?”

##### Self-efficacy

Self-efficacy was measured using four items from the generalized self-efficacy scale (Schwarzer and Jerusalem, 1995). The four statements were rated from 1 (*not at all true*) to 4 (*exactly true*). An example item is: “I am confident that I can deal efficiently with unexpected events.”

##### Performance satisfaction

Performance satisfaction was captured with two self-developed items measuring participants’ satisfaction with the quality of their work. The first item reads: “How satisfied are you with the quality of your work?” The second item measures their satisfaction with achieving objectives: “How satisfied are you with the achievement of the goals you have set for your work?” The correlation between the two items was *r* = 0.66 (*p* < 0.001) for employees and *r* = 0.67 (*p* < 0.01) for coworkers. Answers were scored on a scale ranging from *not at all* (1) to *extremely satisfied* (5, respectively 7 in Study 2).

##### Characteristics of the coworking sample

Since research on people working in coworking spaces is rare, we formulated questions to provide detailed information on the coworking sample we recruited. The questions aimed to collect information on the following topics: a description about the projects coworkers were currently working on (open answer format), the name of the coworking space where participants were working, its location (city), whether this coworking space had a community aspect (yes vs. no), and how long participants had been working in the coworking space (months). We also asked participants how much of their work time they spent in several places (the coworking space, my office, a friend’s office, a home office, other places) and the amount of time they preferred to work in a coworking space (full-time [e.g., 9 to 5, 10 to 6, etc.], for a few hours a day, on weekends, at night, sporadically, other). Finally, we asked them why they chose to work in a coworking space and offered multiple answers (structure in one’s work day, collaboration, flexible working, networking, social interaction, productivity, provision of infrastructure, locational advantages, and cost-efficiency) that had to be rated on a Likert scale from 1 (not important) to 5 (very important).

##### Control variables

Age, gender, and tenure were measured and included in analyses as control variables.

### Results

Analyses of the questions describing characteristics of coworkers showed that the main reason for working at the coworking space was to engage in social interactions (83%). Descriptive statistics on coworkers’ employment status, frequency of coworking space use, occupation, used workplaces, nationality, and reasons for working in a coworking space are presented in **Table 1**. Within **Table 1** we also provide a comprehensive list about projects coworkers are actually working at. Based on the project descriptions one rater determined that coworkers worked primarily in the areas of “software/web development and design” (27%), “consultancy and management” (16%), or “writing, journalism, blogging, and language services” (10%). These categories were validated by a second rater with no information about the study. The second rater assigned the project descriptions to one of these categories (κ = 0.72).

Descriptive statistics, reliabilities (Cronbach’s alphas), and correlations among the study variables for both the employee and coworking samples appear in **Table 2**. In all hypothesis testing analyses, we controlled for age, gender, and tenure, which were not significant in any of the regression models.

**Table 2:** Means, standard deviations, reliabilities (cronbach’s alpha on the diagonal), and correlations between the study variables.

	*M (SD)*	1	2	3	4	5	6
**Employee sample**							
(1) Age	27.66 (3.89)	–					
(2) Gender	1.46 (0.50)	0.11**	–				
(3) Tenure	46.85 (38.14)	0.37***	0.09^∗^	–			
(4) Social support colleagues	3.06 (0.61)	-0.03	-0.07	-0.08	(0.74)		
(5) Self-efficacy	3.18 (0.52)	0.07	0.06	0.10*	0.25***	(0.82)	
(6) Time pressure	3.13 (0.90)	0.04	0.04	0.08	-0.06	0.08^∗^	(0.86)
(7) Performance satisfaction	4.92 (1.26)	0.05	0.07	0.07	0.24***	-0.21^∗∗∗^	0.30^∗∗∗^
**Coworking sample**							
(1) Age	34.86 (8.45)	–					
(2) Gender	1.66 (0.48)	-0.05	–				
(3) Tenure	18.10 (22.31)	0.18*	0.11	–			
(4) Social support coworkers	2.74 (0.58)	-0.21*	0.06	0.09	(0.80)		
(5) Self-efficacy	3.43 (0.43)	0.03	-0.02	0.06	0.10	(0.76)	
(6) Time pressure	2.84 (0.75)	0.11	-0.07	-0.07	-0.01	0.19^∗^	(0.81)
(7) Performance satisfaction	3.93 (0.60)	0.10	-0.08	0.07	0.21*	-0.13	0.17^∗^

*Coworking sample *N* = 154. Employee sample *N* = 609. Gender: 1 = female, 2 = male. *^∗^p* < 0.05, *^∗∗^p* < 0.01, ^∗∗∗^*p* < 0.001*.

To examine the main effect of social support on performance satisfaction (Hypothesis 1), we conducted hierarchical regression analyses. To test mediation (Hypotheses 2a and 2b) and moderated mediation (Hypotheses 3a and 3b), we follow the procedure outlined by Preacher and Hayes (2004). We use an SPSS macro (process) to estimate both mediation and moderated mediation. This stepwise approach estimates indirect effects with both the Sobel test and bootstrapping. Following Becker’s (2005) suggestions, we excluded the control variables from further analyses when they were not significantly correlated with the dependent variable in the regression model. We plotted simple slopes to interpret interaction effects at one standard deviation above and below the mean of the moderator (Aiken and West, 1991). In the coworking sample, we replaced 0.7% (16 out of 2,310) missing values. As recommended by several authors (e.g., Acock, 2005), we ensured that the data were missing completely at random with Little’s Missing Completely at Random (MCAR) test [χ^2^(361, *N* = 154) = 366.30, *p* = 0.41], and we replaced missing data using the expectation-maximization (EM) algorithm in SPSS.

The testing of the postulated hypotheses showed the following: first, we tested the direct effect of social support on performance satisfaction and found a significant positive relationship between these two variables in the employee sample (β = 0.24, *p* < 0.001; Δ*R*^2^ = 0.06, Δ*F* = 37.34, *p* < 0.001) as well as in the coworker sample (β = 0.21, *p* = 0.010; Δ*R*^2^ = 0.04, Δ*F* = 6.80, *p* = 0.010). Results indicate that workers experiencing social support from their colleagues or coworkers are more satisfied with their performance in terms of work quality and achieving objectives. Thus, Hypotheses 1a and 1b are supported.

Second, we tested whether the relationship between social support and performance satisfaction was mediated by self-efficacy (see **Table 3**). For the employee sample, the effects of social support on self-efficacy (path a; β = 0.25, *p* < 0.001) and of self-efficacy on satisfaction with performance (path b; β = 0.26, *p* < 0.001) were significant. Also, the total effect from social support to performance satisfaction was significant (path c: β = 0.24, *p* < 0.001) and remained significant although weakened when the mediator was included (path c’: β = 0.18, *p* < 0.001). These findings were supported by a test of the indirect effect via bootstrapping (95% CI [0.07, 0.21]).

**Table 3:** Results for testing mediation of self-efficacy.

Pathway	Coworking Sample					Employee Sample				
	β	*B*	*SE*	*t*	*P*	β	*B*	*SE*	*t*	*p*
Path a (Social Support -> Self-efficacy)	0.25	0.21	0.03	6.40	<0.001	0.10	0.08	0.06	1.30	0.20
Path b (Self-efficacy –> Performance Satisfaction)	0.26	0.63	0.10	6.54	<0.001	0.15	0.20	0.11	1.81	0.07
Total effect (Path c, Social Support – > Performance Satisfaction)	0.24	0.50	0.08	6.11	<0.001	0.21	0.21	0.08	2.61	0.01
Direct effect (Path c’, Social Support on Performance Satisfaction including Self-efficacy)	0.18	0.36	0.08	4.47	<0.001	0.19	0.19	0.08	2.42	0.01
	
	**Effect**	**Boot SE**	**LLCI**	**ULCI**			**Effect**	**Boot SE**	**LLCI**	**ULCI**
				
Indirect effect (Paths a × b) of Social Support on Performance Satisfaction via Self-efficacy	0.13	0.03	0.07	0.21			0.02	0.02	-0.00	0.08

*Coworking Sample *N* = 154. Employee Sample *N* = 609. LL, lower limit; UL, upper limit; CI, confidence interval; number of bootstraps: 100*.

For the coworking sample, the effect of social support on self-efficacy (path a; *p* = 0.197) and the effect of self-efficacy on satisfaction with performance (path b; β = 0.15, *p* = 0.062) were not significant. The total effect of social support on performance satisfaction (path c) was positive and significant (β = 0.21, *p* = 0.010) and remained significant when self-efficacy was included as a mediator (path c’: β = 0.19, *p* = 0.017). These findings were supported by a test of the indirect effect via bootstrapping (95% CI [-0.00, 0.08]). Thus, these results suggest that self-efficacy mediates the effect of social support on performance satisfaction for employees, but not for coworkers overall, supporting Hypothesis 2a (employee sample), but not Hypothesis 2b (coworking sample).

**FIGURE 2:**
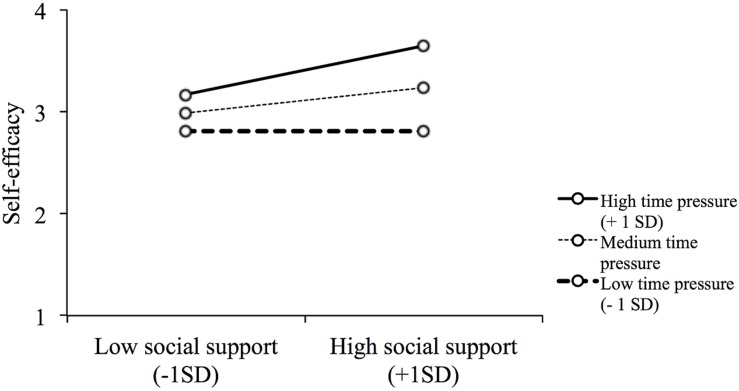
**The moderating effect of time pressure on the relation between social support from coworkers and self-efficacy**.

Finally, we tested whether the mediating effect of self-efficacy was moderated by time pressure according to the analytical approach suggested by Preacher et al. (2007). We do not find such a moderated mediating effect for the employee sample (90% CI [-0.07, 0.05]). However, in the coworking sample, the conditional mediation of self-efficacy was stronger and significant at higher (90% CI [0.01, 0.13]) and medium (90% CI [0.00, 0.07]) levels of time pressure and was weaker and became non-significant at lower levels of time pressure (90% CI = [-0.06, 0.00]). To further clarify this moderating effect, we examined separate simple slopes (Aiken and West, 1991). For the low (*b* = 0.004, *SE* = 0.05, *p* = 0.942) and medium (*b* = 0.081, *SE* = 0.05, *p* = 0.142) time pressure groups, the simple slopes were not significant, whereas the simple slopes for the high (*b* = 0.158, *SE* = 0.06, *p* = 0.005) time pressure group was positive and significant (see **Figure 2**). Thus, Hypothesis 3 was supported in the coworking sample, but not in the employee sample.

### Brief Discussion of Study 2

Findings of Study 2 showed both similar and different effects of social support from colleagues in a traditional office setting and coworkers in a coworking space. First, consistent with previous literature (Searle et al., 2001; Baruch-Feldman et al., 2002; Osca et al., 2005), we found a positive main effect of social support from colleagues and coworkers on satisfaction with performance. Regarding the hypotheses derived from COR theory, we found different effects for employees and coworkers. The proposition of a mediation of self-efficacy (resource gain process) found support only in the employee sample. In the coworking sample, the mediation was only significant in the high time pressure group, providing evidence for the support-mobilization hypothesis (Eckenrode, 1983). In line with previous empirical evidence (Viswesvaran et al., 1999), we found no confirmation for the support-mobilization hypothesis in the employee sample. We conclude that the mobilization of resources seems to be more necessary in coworking spaces than in traditional working contexts.

The main strength of the second study is its inclusion of coworkers working in various coworking spaces across Europe. Moreover, it is the first quantitative multinational study to investigate the effects of social support in coworking spaces. Results are more generalizable than those of previous studies because our study was not limited to one country (see Spinuzzi, 2012) or a small selection of coworking spaces (see Pohler, 2012). However, as the selection of participants is not random, generalizability on the population of coworkers in coworking spaces is limited.

The main limitation of Study 2 concerns the comparability of the two samples. However, the two samples mirror their respective populations well. Another limitation concerns the cross-sectional design, which does not allow for causal inferences. As such, it is impossible to conclude with certainty that social support causes performance satisfaction and not vice versa. However, COR theory suggests a direct effect from the resource social support to consequences. Furthermore, as the data used are based on self-reports, common method bias is possible (Podsakoff et al., 2012). Following recommendations of Podsakoff et al. (2003), we used different response formats and valid scales to reduce possible common method variance. Nevertheless, we did not temporally separate the predictor and criterion. However, since research on coworking spaces is rare, our studies provide valuable information on which to build in the future. Our findings highlight relationships between social support, self-efficacy, and performance satisfaction and emphasize the role of coworking spaces as social ecosystems.

## Discussion on Findings of Studies 1 and 2

Coworking spaces are office environments for independent professionals and are rapidly spreading worldwide. One main reason professionals opt to work in such spaces is the opportunity for social interaction, which diminishes the isolation independent professionals often struggle with (Spinuzzi, 2012). Findings of Study 1 showed that these social interactions come in various forms such as informal social interactions, direct social support (instrumental support, exchange of information), and collaboration. Thus, our findings indicate that coworking spaces are social environments that can provide possibilities for social support with coworkers as a new source of social support. Study 2 showed the effects of this social support and contrasted them with the effects in a traditional work setting. Interestingly, we found a moderated mediation in the coworking sample, but not in the sample of traditional working employees. It seems that a mobilization of support (when time pressure is high) is relevant in the coworking sample. We also found in Study 1 that coworkers engage in various informal social interactions, which can function as a precondition of social support. Engaging in informal social interactions and mobilizing support may cost energy (Norris and Kaniasty, 1996) and should therefore be facilitated by the management of the coworking space.

### Implications for Practice

The findings of the present study underpin the importance of social support in coworking spaces and should encourage coworking spaces to provide the types of contingencies that facilitate social support. Coworking spaces can, for example, display information about other currently present coworkers. Such displays can provide an icebreaker for conversations (Bilandzic et al., 2013). Starting conversations may even be easier when such displays include information about coworkers’ backgrounds, skills, or availability. By such means, coworking spaces can establish interaction as a social norm. Furthermore, we recommend specialization when spaces want to increase social support. Like-minded people or people with similar occupations are more likely to be in similar situations and can consequently better support each other.

At this point, we would like to point out that besides facilitating social support, coworking spaces should also fulfill other needs of coworkers for concentrated work or having dedicated space to perform individual work activities. Quiet rooms in which people can concentrate are as important as group workspaces (Seddigh et al., 2014), especially when social interactions may distract other coworkers.

### Limitations and Suggestions for Future Research

Within the present article, we were able to describe forms of social interactions that happen in coworking spaces. We then investigated effects of one of these forms, social support, but not of the others. Therefore, we encourage further studies to investigate the antecedents and consequences of the two other forms of social interactions in coworking spaces we found in Study 1. These studies may also consider moderating variables such as spatial needs or personal preferences of coworkers. For instance, informal social interactions may diminish feelings of isolation but can also distract coworkers when they need to concentrate on working tasks. Collaboration, on the other hand, may improve productivity of the owned business. Personality variables may also have an impact here. For instance, for people high in extraversion, a first contact may be easier. We also encourage further studies to consider not only social interaction but also aspects of the social atmosphere. For instance, the mere presence of other people can also have an effect on coworkers. Due to social facilitation, simple tasks are performed better in the presence of others (Zajonc, 1965).

The two studies presented within this article represent an important step in describing the social aspect in coworking spaces in general. However, it is still unclear why and under which circumstances coworkers interact with each other. Further studies should deepen the understanding about interactions in coworking spaces. Further studies can, for instance, focus on coworkers’ motives for and personal preferences in interacting with others. Narrative qualitative approaches and analytical approaches that discover aspects below the direct conversation can be useful here to shed light on why coworkers engage in social interactions. It would also be interesting to combine this qualitative approach with network analysis investigating the density and strengths of ties between coworkers in a coworking space and to compare resulting networks with workers in a traditional workplace. Supervisors and colleagues as sources of social support are predefined in traditional workplace settings. In addition, workers may have more superficial or other (private) relationships with other workers of the organization. People working in coworking spaces engage in various forms of social interactions without predefined others to ask for support. Furthermore, feedback and input from other coworkers can be more easily ignored than those from traditional workers. A better understanding of motives, preferences, and networks will enable the implementation of targeted interventions to increase social support in coworking spaces.

The cross-sectional approach of Study 2 is limited because it does not allow causal conclusions. Thus, there is a need for longitudinal studies that examine predictors, activators, and consequences in the context of receiving support in a coworking space. After receiving social support, various effects are possible. For instance, according to the social support deterioration deterrence model (Norris and Kaniasty, 1996), deterioration occurs after a mobilization of resources. Therefore, we can also think of negative effects such as emotional exhaustion in the long term. There may also be differences between traditional office workers and coworkers. Coworkers may have to put more effort in activating support, while at the same time they can ignore feedback more easily than workers can from colleagues and supervisors. We further recommend for these studies to rely on data that are not solely self-reported to avoid common method bias. For instance, social support can be assessed from different sources, or objective or physiological data about emotional exhaustion can be included.

## Concluding Remarks

In the last couple of years, increasing numbers of independent professionals have opted to work in coworking spaces. This emerging office type appears to provide a resourceful environment for this particular target group because it provides opportunities for social support in addition to flexible business infrastructure. To date, only a few scientific investigations of coworking spaces have been conducted. Our second study is, to the best of our knowledge, the first to quantitatively investigate social support in coworking spaces across Europe. The findings highlight the importance of coworkers as a source of social support among independent professionals and should encourage studies that further explore coworking spaces as a social office environment likely to grow even more in the future.

## Author Contributions

CG, TES, and CK designed Study 1; CG, JA, and CK designed Study 2; CG and JA conducted research; CG, JA, and TES analyzed the data; CG wrote the article; TES edited the article; All authors read and edited a draft of the final article.

## Conflict of Interest Statement

The authors declare that the research was conducted in the absence of any commercial or financial relationships that could be construed as a potential conflict of interest.

## Footnotes

1 wiki.coworking.org.

2 www.respondi.com

3 wiki.coworking.org
